# Response to the Comment on “Development and Validation of a Machine Learning‐Based Marker for Early Detection and Prognosis Stratification of Nonalcoholic Fatty Liver Disease”

**DOI:** 10.1002/advs.202518968

**Published:** 2025-11-10

**Authors:** Lushan Xiao, Lin Zeng, Jiaren Wang, Chang Hong, Li Liu

**Affiliations:** ^1^ Department of Health Management Nanfang Hospital Southern Medical University Guangzhou 510515 China; ^2^ Guangdong Provincial Key Laboratory of Viral Hepatitis Research Department of Infectious Diseases Nanfang Hospital Southern Medical University Guangzhou 510515 China; ^3^ Department of Gastroenterology Shenzhen Hospital Southern Medical University Shenzhen 518133 China

We sincerely thank Dr. Peng and Dr. Duan for their insightful and constructive commentary on our recent study concerning a machine learning‐based marker for the early detection and prognostic stratification of nonalcoholic fatty liver disease (NAFLD).^[^ ^1^ ^]^ Their comments raise several pivotal questions that are vital for the continued development and future clinical implementation of non‐invasive tools for NAFLD. We appreciate the opportunity to further clarify our study, provide additional context and data, and contribute to a deeper understanding of the potential role of the “in‐silico score for NAFLD (ISNLD)” model in clinical practice.

Regarding the comparative analysis of different models, we first need to clarify that the design of the ISNLD model has a dual purpose. The primary objective of ISNLD is to effectively identify populations at high risk for NAFLD. And the more critical and clinically significant aim is to precisely stratify the long‐term prognostic risks of disease progression to advanced liver disease (such as cirrhosis and hepatocellular carcinoma) and extrahepatic adverse outcomes among these individuals. In the clinical management of NAFLD, progression to significant fibrosis is an important factor in both liver‐related and all‐cause mortality. Consequently, the clinical utility of a predictive model is defined by its ability to forecast disease progression. The Fibrosis‐4 (FIB‐4)^[^ ^2^ ^]^ index was explicitly developed for this purpose and is recommended by multiple international guidelines—including those from the Chinese Society of Hepatology,^[^ ^3^ ^]^ the American Association for the Study of Liver Diseases (AASLD),^[^ ^4^ ^]^ and the European Association for the Study of the Liver (EASL)^[^ ^5^ ^]^—as a first‐line non‐invasive test for risk stratification of advanced fibrosis. It represents the established standard of care for prognostic assessment in current clinical practice. Given this clinical context, we selected FIB‐4 as the comparator to evaluate the risk stratification capability of our model.

Furthermore, to address its screening utility, we also compared our model against the NAFL Screening Score (NSS), a user‐friendly scoring tool designed for NAFLD detection. In comparison with the Framingham Steatosis Index (FSI), Hepatic Steatosis Index (HSI), and Fatty Liver Index (FLI), NSS demonstrates the highest sensitivity and the area under the curve (AUC).^[^ ^6^ ^]^ As detailed in **Table** **1**, our developed model significantly outperformed NSS across all key performance metrics, including AUC, accuracy, sensitivity, and specificity.

**Table 1:** Performance metrics of the stacked model and traditional indicators for predicting NAFLD.

	AUC (95%CI)	Accuracy (95%CI)	Sensitivity (95%CI)	Specificity (95%CI)
**Train set**				
Stacked Model	0.843 (0.838,0.848)	0.752 (0.747,0.757)	0.788 (0.779,0.796)	0.736 (0.730,0.742)
FIB‐4	0.534 (0.526,0.541)	0.674 (0.669,0.679)	0.040 (0.036,0.044)	0.975 (0.973,0.977)
NSS	0.688 (0.681,0.694)	0.618 (0.612,0.624)	0.689 (0.680,0.698)	0.584 (0.577,0.591)
**Internal test set**				
Stacked Model	0.840 (0.831,0.849)	0.763 (0.753,0.773)	0.757 (0.740,0.775)	0.765 (0.753,0.777)
FIB‐4	0.532 (0.518,0.547)	0.662 (0.651,0.673)	0.065 (0.055,0.075)	0.955 (0.950,0.961)
NSS	0.693 (0.681,0.706)	0.664 (0.653,0.675)	0.601 (0.581,0.621)	0.695 (0.682,0.708)
**External test set**				
Stacked Model	0.872 (0.864,0.880)	0.777 (0.768,0.786)	0.830 (0.815,0.846)	0.760 (0.749,0.770)
FIB‐4	0.543 (0.529,0.557)	0.527 (0.517,0.537)	0.553 (0.533,0.574)	0.519 (0.507,0.531)
NSS	0.768 (0.757,0.779)	0.683 (0.673,0.693)	0.739 (0.721,0.757)	0.664 (0.653,0.676)

Abbreviation: AUC, Area under the curve, FIB‐4, Fibrosis‐4 Index; NSS, NAFL screening score.

And we concur with the point that abdominal ultrasonography has inherent limitations in diagnosing hepatic steatosis, particularly its limited sensitivity in detecting mild steatosis. Liver biopsy, the diagnostic gold standard, is unsuitable for large cohort studies or screening due to the invasiveness, potential for complications, and sampling error. Meanwhile, more accurate imaging techniques like Magnetic Resonance Imaging Proton Density Fat Fraction (MRI‐PDFF), while excellent for quantifying liver fat, are prohibitively expensive and have limited accessibility, precluding their use in routine clinical screening. Both the EASL^[^ ^5^ ^]^ and the Asia‐Pacific Association for the Study of the Liver (APASL)^[^ ^7^ ^]^ guidelines recommend ultrasound as the first‐line imaging modality for NAFLD screening, owing to its advantages in availability, safety, and cost‐effectiveness. Given that the health examinee dataset of Nanfang Hospital was a large‐scale research cohort based on a general population undergoing health check‐ups, ultrasonography represents a highly suitable and practical method for assessing hepatic steatosis in this setting. Recognizing the importance of this issue, we plan to conduct relevant analyses to evaluate the impact of ultrasound as an assessment method for fatty liver on the model and endeavor to collect MRI‐PDFF data to further validate our model's performance in the future, which may provide a more comprehensive understanding of the utility of ISNLD across different diagnostic settings.

Another crucial point concerns the challenges of applying a model trained on a European‐ancestry cohort to a Chinese population. Genetic models derived from European data often exhibit a significant drop in predictive performance when applied to non‐European populations due to differences in genetic architecture, allele frequencies, and linkage disequilibrium (LD) patterns. In this study, we did not utilize advanced statistical genetics methods—such as PRS‐CSx or multi‐ethnic fine‐mapping—to adjust single nucleotide polymorphisms (SNP) weights, partly driven by the concern that such population‐specific optimization could reduce the model's broader generalizability. Despite the absence of specific genetic optimization, the ISNLD model maintained strong predictive performance in the Chinese cohort. We believe this result strongly indicates an inherent robustness in the model's architecture, which is a promising feature for its potential global applicability.

Finally, we wish to address the practical concerns regarding the model's complexity and its feasibility for large‐scale clinical application. In clinical practice, there is a well‐known tension between a model's predictive accuracy and the ease of use. Oversimplified models, while easy to calculate, often suffer from lower accuracy, which can lead to critical misclassification errors. A certain level of model complexity is necessary to achieve the high degree of precision required for meaningful early detection and prognosis stratification. The superior prognostic power of ISNLD is derived directly from its comprehensive feature set, particularly the integration of a robust genetic dataset, which justifies the increased data requirement.

In terms of cost, we used the Infinium Chinese Genotyping Array (CGA) v1.0 BeadChip to conduct whole‐genome genotyping in the health examinee dataset of Nanfang Hospital, with the array being a genotyping platform developed by Illumina specifically tailored for Chinese populations. A single microarray can simultaneously genotype 684023 SNPs across 24 samples, with a testing cost of approximately ¥280 yuan ($38.4) per sample. Compared to computed tomography (CT) or magnetic resonance imaging (MRI) examinations, this approach offers a more cost‐effective solution for NAFLD detection. And we selected 217 SNPs significantly associated with NAFLD from a vast array of genomic loci. Implementation of these SNPs as a targeted panel in the large‐scale population screening may achieve improved convenience and cost‐effectiveness. Moreover, to minimize the burden on clinicians, we envision creating a user‐friendly, web‐based ISNLD calculator or integrating the model into Electronic Health Record (EHR) systems to streamline its application.

In summary, we thank Dr. Peng and Dr. Duan for their thoughtful comments again, which have allowed us to strengthen and clarify our work. We believe that addressing these points will enhance the value of our work for the scientific community and ultimately contribute to better screening and management strategies of NAFLD. We are committed to advancing this research and welcome further discussion on the implementation of machine learning approaches in NAFLD detection and prognosis.

## Conflict of Interest

The authors declare no conflict of interest.
